# The Relative Influences of Phosphometabolites and pH on Action Potential Morphology during Myocardial Reperfusion: A Simulation Study

**DOI:** 10.1371/journal.pone.0047117

**Published:** 2012-11-07

**Authors:** Byron N. Roberts, David J. Christini

**Affiliations:** Greenberg Division of Cardiology and Department of Physiology, Biophysics and Systems Biology, Weill Cornell Medical College, New York, New York, United States of America; University of Minnesota, United States of America

## Abstract

Myocardial ischemia-reperfusion (IR) injury represents a constellation of pathological processes that occur when ischemic myocardium experiences a restoration of perfusion. Reentrant arrhythmias, which represent a particularly lethal manifestation of IR injury, can result when ischemic tissue exhibits decreased excitability and/or changes of action potential duration (APD), conditions that precipitate unidirectional conduction block. Many of the cellular components that are involved with IR injury are modulated by pH and/or phosphometabolites such as ATP and phosphocreatine (PCr), all of which can be manipulated *in vivo* and potentially in the clinical setting. Using a mathematical model of the cardiomyocyte that we previously developed to study ischemia and reperfusion, we performed a series of simulations with the aim of determining whether pH- or phosphometabolite-related processes play a more significant role in generating changes in excitability and action potential morphology that are associated with the development of reentry. In our simulations, persistent shortening of APD, action potential amplitude (APA), and depolarization of the resting membrane potential were more severe when ATP and PCr availability were suppressed during reperfusion than when extracellular pH recovery was inhibited. Reduced phosphometabolite availability and pH recovery affected multiple ion channels and exchangers. Some of these effects were the result of direct modulation by phosphometabolites and/or acidosis, while others resulted from elevated sodium and calcium loads during reperfusion. In addition, increasing ATP and PCr availability during reperfusion was more beneficial in terms of increasing APD and APA than was increasing the amount of pH recovery. Together, these results suggest that therapies directed at increasing ATP and/or PCr availability during reperfusion may be more beneficial than perturbing pH recovery with regard to mitigating action potential changes that increase the likelihood of reentrant arrhythmias.

## Introduction

When myocardium becomes ischemic and subsequently experiences a return of normal perfusion, pathological changes can occur that culminate in a collection of phenomena known as ischemia-reperfusion (IR) injury. IR injury includes mechanical pump failure, increased cell death, and cardiac arrhythmias [1], [2]. With regard to arrhythmias, reentry is a condition in which a region of the myocardium is rapidly and repeatedly activated, and is believed to be a significant cause of arrhythmias in the ischemic setting [3]. Two of the lethal arrhythmias that can result from IR injury — ventricular tachycardia and fibrillation — are reentrant arrhythmias. Unidirectional conduction block is required for the initiation of reentry [3], as it allows for the possibility of retrograde conduction in one limb of a reentrant circuit. Several mechanisms have been proposed for the creation of unidirectional block [4], including heterogeneities in tissue excitability and spatial dispersion of refractoriness. In turn, a spatial dispersion of refractoriness can be increased by premature stimuli, action potential duration (APD) shortening, action potential duration alternans (which can be induced by calcium transient alternans [5]), and nonuniform cellular coupling [6]. Ischemic tissue commonly exhibits elevated RMP and APD shortening that is induced by a variety of processes [7].

**Figure 1:**
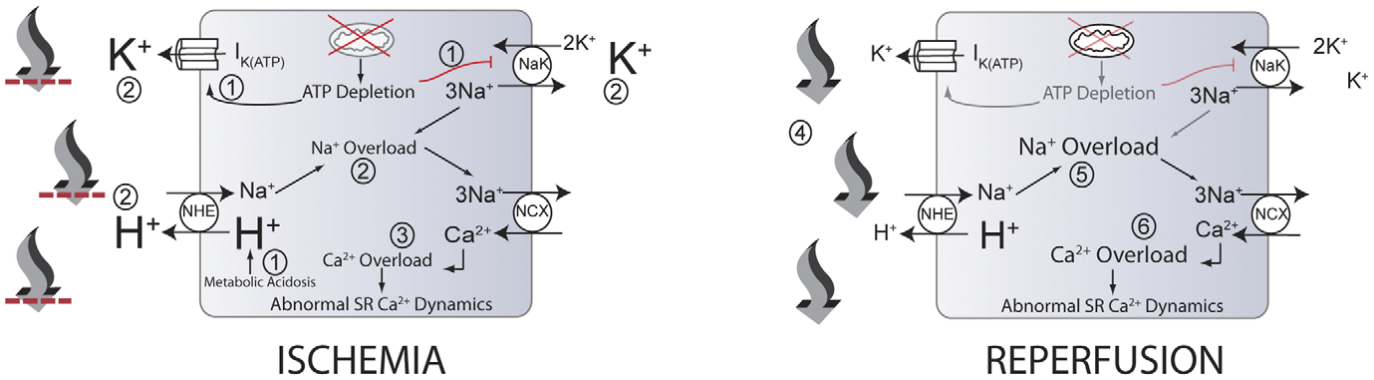
Some events that occur during myocardial ischemia and reperfusion. During ischemia, ATP depletion leads to inhibition of the sodium-potassium pump (NaK) and increased efflux through the ATP-regulated potassium channel (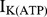) (1). Also, increased anaerobic metabolism produces a metabolic acidosis (1). Increased 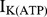 and decreased NaK flux contribute to the accumulation of extracellular potassium (2) (larger font). In addition, intracellular acidosis drives increased flux through the sodium-proton exchanger (NHE), contributing to extracellular acidosis (larger font) and intracellular sodium accumulation (2), worsened by decreased NaK flux. Increased intracellular sodium results in the sodium-calcium exchanger (NCX) operating more in the reverse mode, contributing to increased myoplasmic calcium concentration (3). High intracellular calcium concentrations can lead to abnormal sarcoplasmic reticulum calcium cycling and proarrhythmic phenomena. Upon reperfusion, washout of acidotic, hyperkalemic extracellular fluid occurs (4), reducing the concentrations of extracellular potassium and protons (smaller font). The resulting proton gradient allows increased flux through the NHE, resulting in exacerbations of intracellular sodium (5) and calcium (6) overloads (larger font) and additional proarrhythmic phenomena. Note that numbers in this legend correspond to encircled numbers in figure, not references. Figure and legend reproduced from [25].

Many of the changes that occur during ischemia and reperfusion are illustrated in Fig. 1. These changes include development of acidosis and subsequent restoration of a proton gradient during reperfusion [1], [2], [8], depletion of phosphometabolites including ATP and phosphocreatine (PCr) [7], [9], accumulation of extracellular potassium (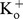) and subsequent washout during reperfusion [10], elevated loads of intracellular sodium (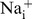) and calcium (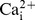) [2], [11], and inhibition of many cell components by acidosis and loss of phosphometabolite availability.

These processes have multiple implications for the initiation of reentry via direct modulation of transmembrane currents and altered ion homeostasis. Elevated [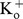] can depolarize the RMP, suppressing cell excitability and modulating ion channel currents through altered ionic gradients [7]. 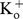 accumulation is largely a result of impaired NaK function (secondary to reduced ATP and PCr availability, as well as acidosis) [7], [12]. Increased [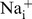] is also a sequela of impaired NaK function, in addition to increased sodium-proton exchange during acidosis [2]. Elevated [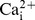], mediated by elevated [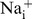] [2], can result in calcium transient alternans [13] (which can manifest as APD alternans), as well as spontaneous calcium releases [14], which can lead to the generation of premature stimuli. Inhibition of SERCA [15]–[18] and ryanodine receptor release [15], [19] by suppressed pH and/or ATP availability can also have implications for the development of calcium transient alternans. Sodium-calcium exchange (NCX), which is a primary exporter of calcium from the cell, is impaired under acidic conditions [7], [15], [16], [20]. Finally, APD and dispersion of refractoriness can be modulated by changes in 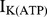, 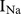, and 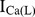 flux, which in turn are affected by changes in ion concentrations, pH, and phosphometabolite availability [7], [21].

Perturbations to pH and phosphometabolite status should be attractive targets for the mitigation of ischemia-reperfusion injury, as they are relatively accessible. pH can be controlled by varying carbon dioxide partial pressure and/or bicarbonate, both routinely manipulated in the clinical setting. Preclinical experiments aimed at inhibiting pH recovery by reperfusing myocardium with an acidic buffer [1], [22], [23] have been performed, with variable success. Increasing ATP availability by delivering liposomes containing ATP into ischemic myocardium has been investigated as a therapeutic option [24].

Given that these accessible therapeutic targets affect multiple cellular components — in sometimes complex ways — to produce pathology in the setting of ischemia and reperfusion, we sought to use a mathematical model to yield additional insight regarding their roles. We first posed the question of whether pH- or phosphometabolite-related alterations play a more significant role in generating the changes to action potential morphology that are associated with the initiation of reentrant arrhythmias. We then sought to identify some of the links between impaired pH and phosphometabolite status and altered transmembrane currents that produce these action potential changes. Our results suggest that impaired phosphometabolite status during reperfusion plays a more significant role than impaired pH recovery in persistent APD and APA reduction throughout reperfusion. Changes to action potential morphology during reperfusion are caused by direct modulation of ion channels and exchangers, as well as indirect consequences of elevated sodium and calcium load during reperfusion.

## Methods

### Mathematical Model

**Figure 2:**
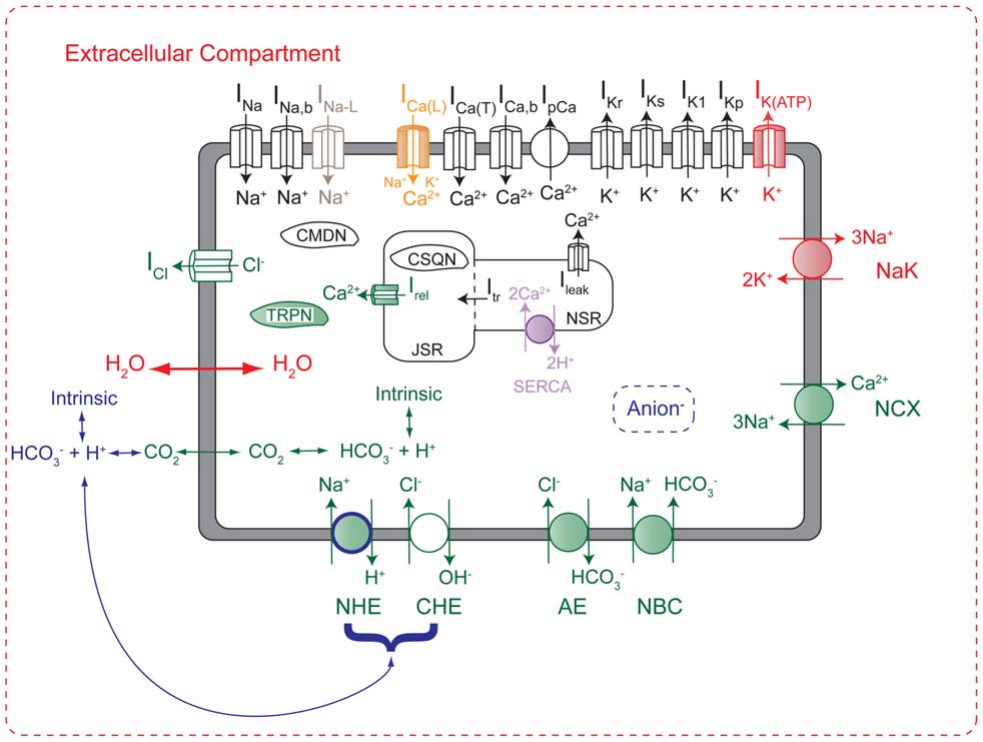
Mathematical model schematic. The model is an LRd model (black), with additions and modifications that were implemented from the CS (green) [15] and TCS [12] models (red), as well as an improved SERCA pump from [18] (purple), late sodium current from [27] (grey), the implementation of ATP-modified L-type calcium channel availability from [21] (orange), and novel additions by the authors (blue). Shaded components represent those that are regulated by pH and/or phosphometabolites. The model includes systems of equations that regulate concentrations of ATP, ADP, AMP, inorganic phosphate, creatine, and phosphocreatine, as well as impermeant metabolites that affect water flux. Abbreviations: Anion: generic anion species produced during ischemia; NCX, sodium-calcium exchanger; NHE, sodium-hydrogen exchanger; NBC, sodium-bicarbonate symporter; CHE, chloride-hydroxide exchanger; AE, anion exchanger; CMDN, calmodulin; TRPN, troponin C; CSQN, calsequestrin; JSR, junctional sarcoplasmic reticulum; NSR, network sarcoplasmic reticulum. Figure and legend reproduced from [25].

The mathematical model used for these simulations was described in detail in a recently published paper [25]. This predominantly guinea pig cardiomyocyte model, shown in Fig. 2, contains representations of many cell components (shaded in Fig. 2) that are sensitive to changes in pH and/or the phosphometabolites phosphocreatine (PCr), ATP, ADP, and AMP. The model has been validated against a wide range of experimental data types and sources, the details of which can be found in [25]. Integration was performed using the forward Euler method with a time step of 0.005 ms. The model was initialized with the conditions listed in Table 1. For all simulations, the cell was constantly stimulated using pulses of −80.0 uA/uF lasting 0.5 ms.

**Table 1:** Initial Conditions.

Parameter	Value
V	−77.00
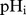	7.15
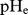	7.40
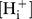	0.000070795
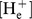	0.000039811
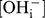	0.000141254
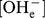	0.000251189
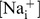	14.34
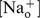	149.02
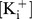	109.10
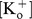	5.40
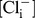	33.77
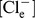	146.7
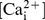	0.000335
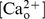	1.80
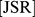	0.605236
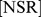	2.352399
m	0.005751
h	0.894526
j	0.889565
d	0.000022
f	0.990176
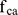	0.640341515
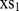	0.059092
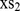	0.108548
xr	0.005171
b	0.002925
g	0.899522
p	0.018649
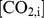	0.741684
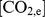	0.741684
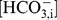	7.90
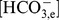	11.90
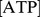	7.216
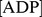	0.036
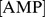	0.000184
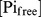	0.8
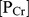	13.3
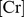	8.9
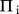	310.0
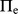	310.0
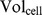	0.00003801
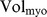	0.000025847
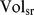	0.000002281
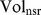	0.000002098
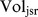	0.000000182
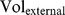	0.000005172
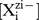	0.00393598
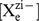	0.000036618

### Simulation Protocols

**Figure 3:**
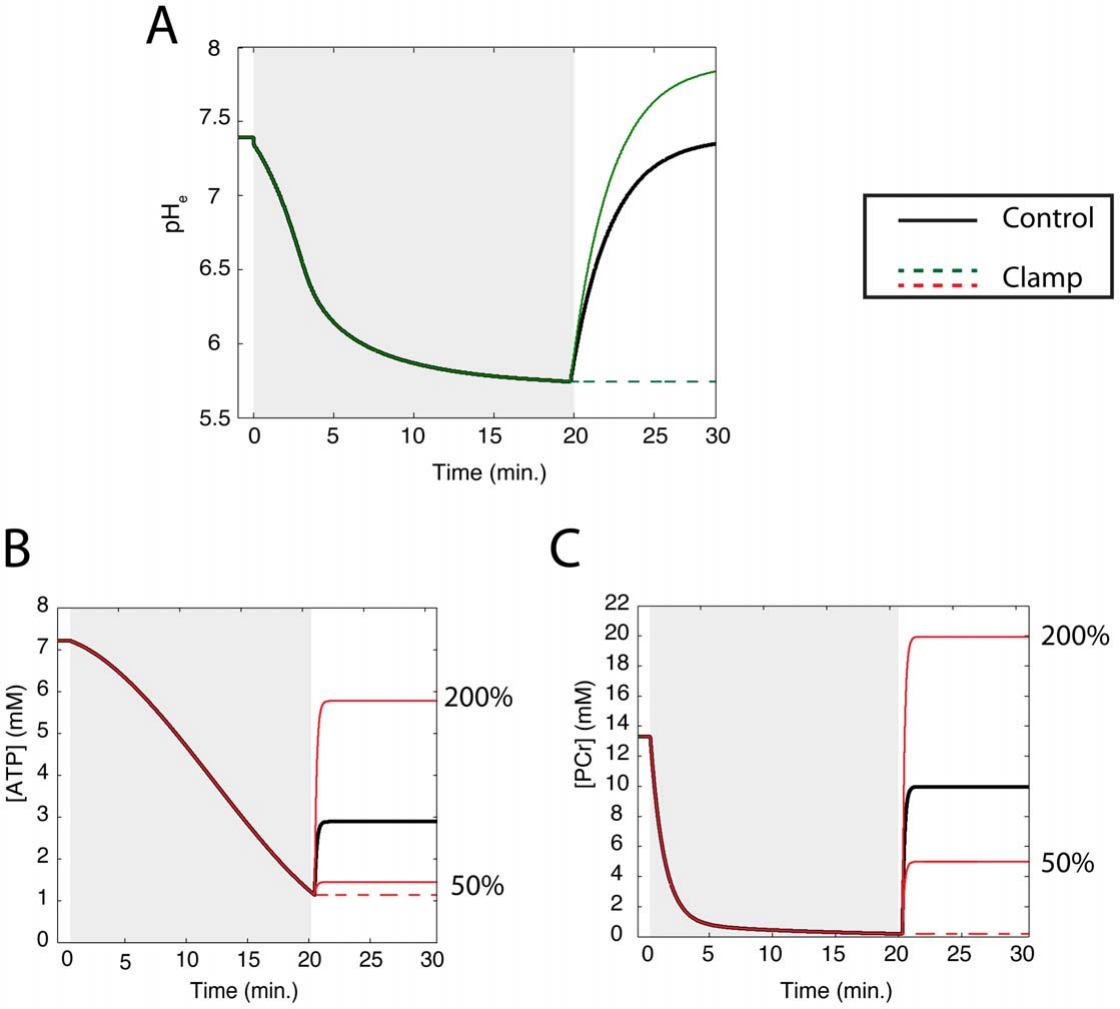
Summary of simulations. In each simulation, the end-reperfusion targets of extracellular pH (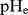) (A) and/or ATP (B) and PCr (C) were controlled. Gray regions denote ischemic phase of simulations. In the control simulation (solid black lines), 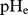 recovered toward a target during reperfusion that was equal to its preischemic value, while ATP and PCr concentrations recovered to values that were 40 and 75 percent of their preischemic concentrations, respectively. In the Dual Clamp simulation, 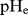 and the concentrations of ATP and PCr were held at their end ischemic values throughout the 10 minutes of reperfusion. In the “pH” simulations, 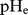 was varied during reperfusion while ATP and PCr concentrations followed their control protocols (solid black lines). In the “pH Clamp” simulation (dashed green line in (A)), 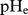 was held at its end ischemic value throughout reperfusion. In the “pH 7.9” simulation (solid green line in (A)), 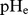 recovered toward a value of 7.9. Conversely, in the “ATP” simulations, the recovery protocols for ATP and PCr were varied while 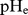 followed its control protocol. In the “ATP Clamp” simulation (dashed red lines in (B) and (C)), the concentrations of ATP and PCr were held at their end ischemic values throughout reperfusion. In the “ATP 50” and “ATP 200” simulations (solid red lines in (B) and (C)), the concentrations of both ATP and PCr recovered to 50 and 200 percent of their targets in the control simulation, respectively. This represents only a subset of the simulations that were performed. The full simulation set is summarized in Text S1.

In order to explore the effects of pH and phosphometabolite concentrations during reperfusion, we controlled the end-reperfusion targets of extracellular pH (which significantly affects intracellular pH recovery) and/or ATP and PCr during simulated reperfusion. In the control simulation, extracellular pH (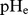) recovers toward a target value of 7.4 (Fig. 3A (black line)), as described by the following equation:
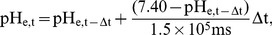
(1)where 7.40 is the pre-ischemic 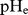 and 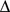
t is the length of the time step.

Also, the control simulation protocol dictates that ATP and PCr concentrations target end-reperfusion values equal to 40 and 75 percent of their preischemic values, respectively (Figs. 3B and C, respectively (black lines)), as described by the following equations:
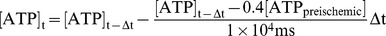
(2)

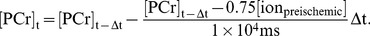
(3)


In simulations examining the role of pH, 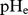 was either clamped at its end-ischemic value (green dashed line in Fig. 3A), or allowed to recover toward a value of 7.9 (green solid line). In each of these simulations, ATP and PCr concentrations were allowed to recover as they do in the control scenario (black lines in Figs. 3B and 3C, respectively).

In a separate series of simulations, we allowed 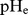 to follow its control course (black line in Fig. 3A), but varied the target concentrations of ATP and PCr. These concentrations were either clamped at their end-ischemic values (dashed lines in Figs. 3B and C), or targeted to 50 or 200 percent of their control recovery targets (solid red lines). We also performed a simulation in which all three parameters were clamped at their end-ischemic values, for a total of 7 simulations. In figures and tables, “ATP” refers to both ATP and PCr modifications, as the end-reperfusion targets of these two metabolites were changed in unison.

In order to more fully explore how the system responds to perturbations of 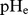 and phosphometabolite availability, we performed five additional simulations. In two simulations, 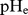 recovery targets were set at 6.4 and 6.9 while ATP and PCr recovered as in the control simulation. In three additional simulations, ATP and PCr concentration targets were set to 25, 75, and 150 percent of their control values (while pH recovered as in the control simulation). For ATP, it should be noted that targeting 25 percent of the control value further decreases the concentration. This full set of 12 simulations is summarized in Text S1, where the results can also be found.

The aforementioned simulations were performed using a pacing rate of 3 Hz, and unless otherwise specified, we refer to simulations using this rate throughout the remainder of the manuscript.. However, in order to ensure that our conclusions were not dependent upon pacing rate, we repeated the seven simulations summarized in Fig. 3 at pacing rates of 2 and 4 Hz.

It should be noted that when ATP and PCr concentrations are clamped, the concentrations of other phosphometabolites may still change. ADP is dependent upon creatine (Cr), which is calculated directly from PCr and intracellular proton concentrations. In turn, AMP concentration is updated based upon concentrations of ADP and ATP. The concentration of free inorganic phosphate is also dependent upon concentrations of ATP, ADP, AMP and PCr. Thus, even when ATP and PCr concentrations are held constant, intracellular pH will have an effect on other phosphometabolite concentrations. The equations that dictate these phosphometabolite concentrations are provided in Text S1.

## Results

### Action Potential Morphology

**Figure 4:**
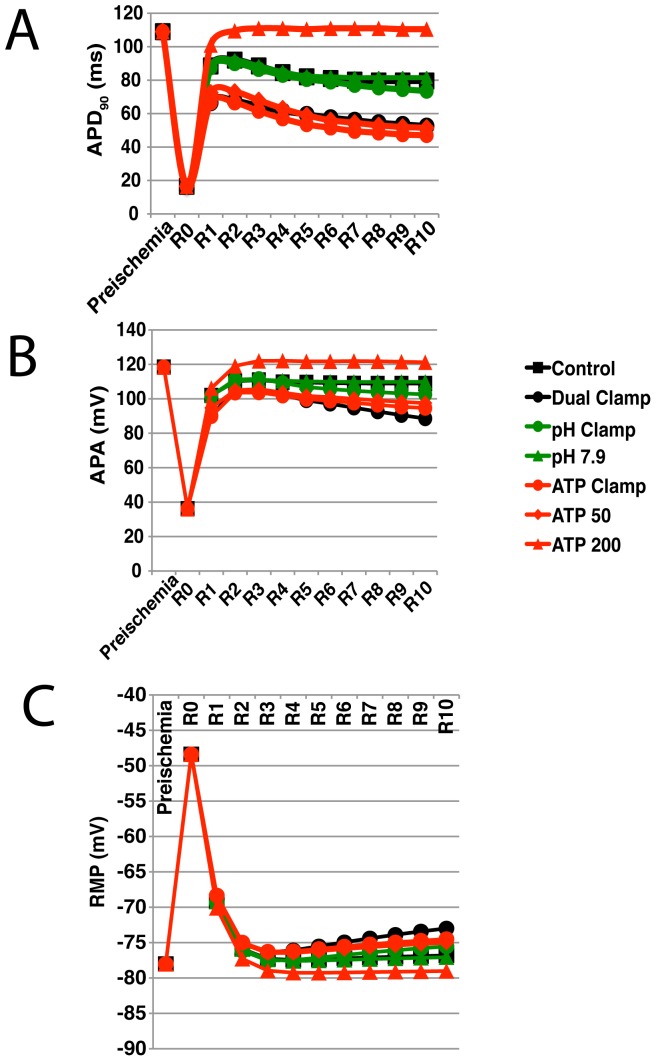
Action potential duration (90), action potential amplitude, and resting membrane potential during simulated reperfusion. The evolution of action potential duration (90) (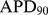) (A), action potential amplitude (APA) (B), and resting membrane potential (RMP) (C) throughout reperfusion are shown for the subset of simulations shown in Fig. 3. Values recorded at the end of preischemia, at the beginning of reperfusion (R0), and once per minute of reperfusion (R1–R10) are shown. Control and Dual Clamp simulations are represented by black squares and circles, respectively. pH Clamp and pH 7.9 simulations are represented by green circles and triangles, respectively. ATP Clamp, ATP 50, and ATP 200 simulations are represented by red circles, diamonds, and triangles, respectively. Simulations were performed using a pacing rate of 3 Hz. Results for the full set of 3 Hz simulations can be found in Text S1.

**Figure 5:**
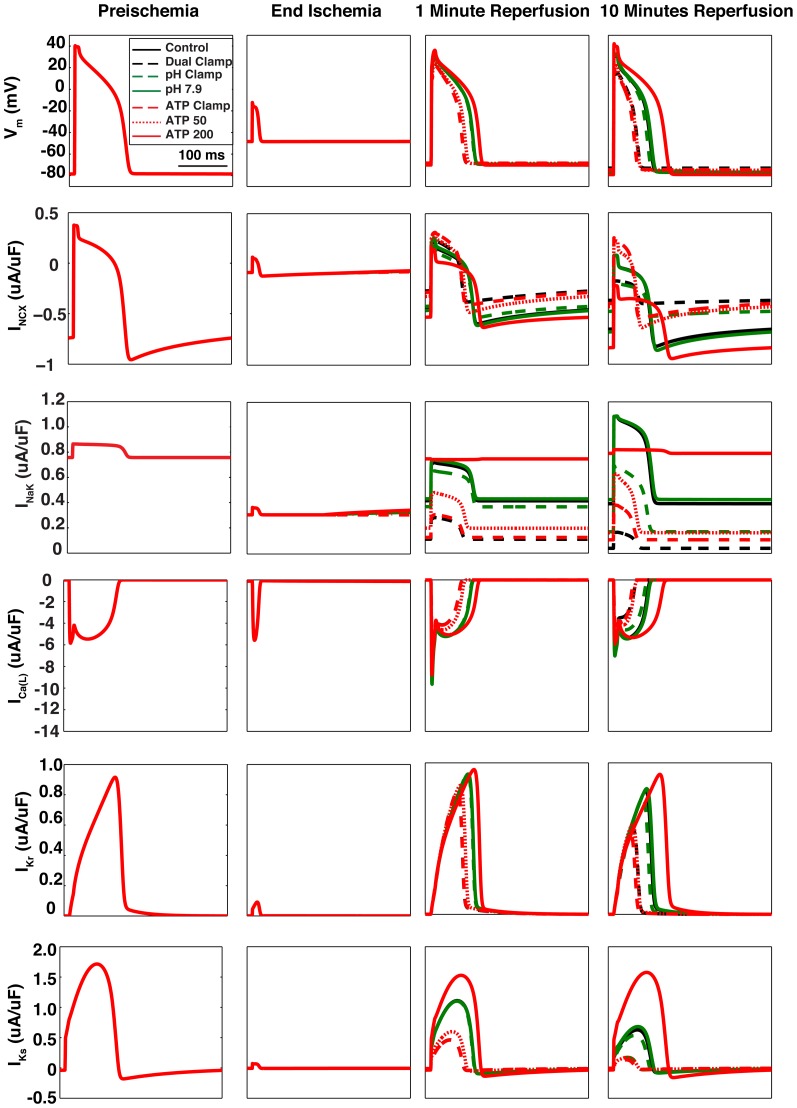
Representative action potentials and current traces. Action potentials and current traces for NCX, NaK, 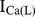, 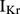, and 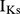 were recorded just before the onset of ischemia, at the end of ischemia, after 1 minute of reperfusion, and after 10 minutes of reperfusion. Each panel represents 333 ms (the cycle length used in these simulations). Within each row, the scales of all four panels are shown to the far left. Control and Dual Clamp simulations are represented by black solid and dashed lines, respectively. pH Clamp and pH 7.9 simulations are represented by green dashed and solid lines, respectively. ATP Clamp, ATP 50, and ATP 200 simulations are represented by red dashed, dotted, and solid lines, respectively. For panels in the first two columns, all lines are superimposed, as the simulation protocols differed only after the onset of reperfusion.

The evolution of action potential durations throughout reperfusion for each simulation are provided in Fig. 4A. Representative action potentials are shown in Fig. 5. Inhibiting 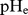 recovery produces only small changes in APD relative to control (black squares are strongly overlapped by both sets of green lines in Fig. 4). Interestingly, the mean APD was slightly shorter when 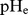 was allowed to recover slightly to 6.4 (see Text S1).

On the other hand, phosphometabolites played a much more significant role in the evolution of action potential duration during reperfusion. In particular, most of the effects seen in the Dual Clamp simulation appear to be caused by reductions in ATP and PCr availability (strong overlap between black circles and red circles in Fig. 4). Also, there is a clear relationship between the amount of ATP and PCr made available during reperfusion and APD. It was noted that unless more ATP and PCr were made available than during the control simulation, APD would increase over the first two minutes of reperfusion, then fall toward a lower steady state value.

In Fig. 4B, action potential amplitudes (APA) are reported for every minute of reperfusion for each simulation, along with the starting preischemic value. As was the case for APD, phosphometabolites appear to play a bigger role in restoring normal amplitude than does pH. For both pH and phosphometabolites, there was a direct relationship between the end-reperfusion target values and mean APA. Also, as with APD, in many simulations there was an initial increase over the first two minutes of reperfusion, followed by a decrease. The presence and severity of this decrease depended on how much ATP and PCr were made available during reperfusion (red lines), or to a lesser degree, the amount of pH recovery (green lines).

The evolution of resting membrane potential (RMP) throughout reperfusion in each simulation is presented in Fig. 4C. Again, phosphometabolite availability during reperfusion appears to play a bigger role than does the extent of pH recovery. There are straightforward relationships between RMP during reperfusion and pH recovery and phosphometabolite availability. These relationships are mediated by intracellular potassium, which as discussed later, depends on NaK function.

**Figure 6:**
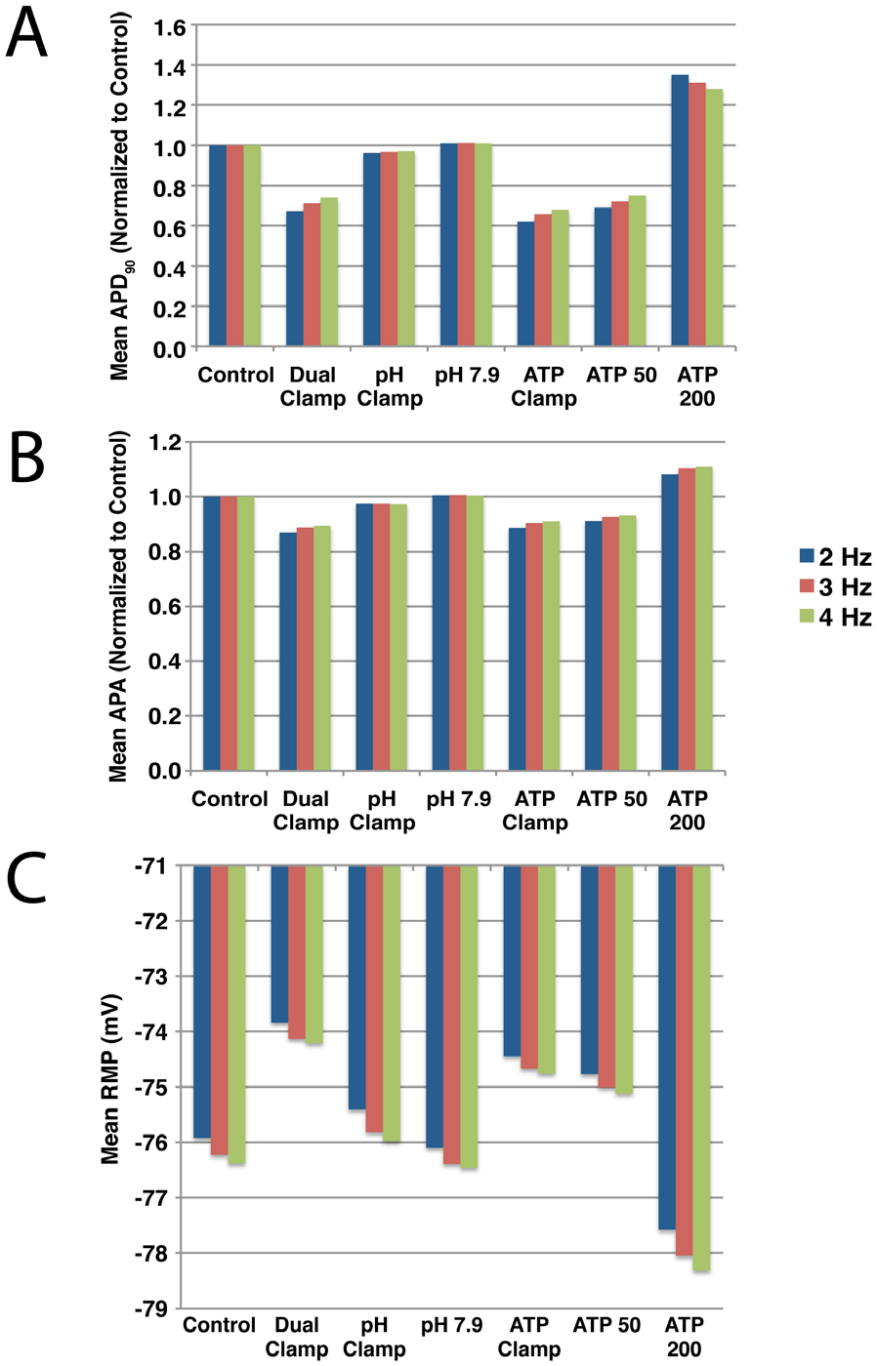
Mean 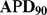, APA and RMP during reperfusion. Each of the seven simulation protocols summarized in Fig. 3 were run at three pacing rates: 2 Hz (blue), 3 Hz (red), and 4 Hz (green). For each simulation, the mean APD (A), APA (B), and RMP (C) were calculated for the 10 minutes of reperfusion. For each pacing rate, the mean 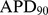 and APA values are normalized to the control simulation. Mean values for the full set of simulations that were run using a pacing rate of 3 Hz can be found in Text S1.

Fig. 6 presents the mean APD, APA, and RMP during reperfusion for each of the seven simulations summarized in Fig. 3 at three different pacing rates. Action potential characteristics and ion homeostasis are all rate-dependent, so differences would be expected between pacing rates. However, despite these differences, the trends across the seven pacing protocols were the same for all three pacing rates. The shortest action potential durations and amplitudes were observed in the Dual and ATP Clamp protocols, regardless of pacing rate, while the largest durations and amplitudes were observed in the ATP 200 protocol. Similarly, RMP was most depolarized in the Dual Clamp simulations, regardless of pacing rate, while hyperpolarization was greatest with the ATP 200 protocol.

In summary, the extremes of action potential morphology were associated with the most extreme perturbations to ATP and PCr availability during reperfusion. Alterations to action potential morphology differ relatively little from control whether 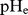 is clamped at its end-ischemic value or forced to an even higher value than control during reperfusion. In contrast, changes in action potential morphology when ATP and PCr availability are clamped at end-ischemic values are similar to those seen when both pH and phosphometabolites are clamped (Dual Clamp). These observations suggest that phosphometabolite status plays a larger role than pH during reperfusion.

### Transmembrane Currents

**Figure 7:**
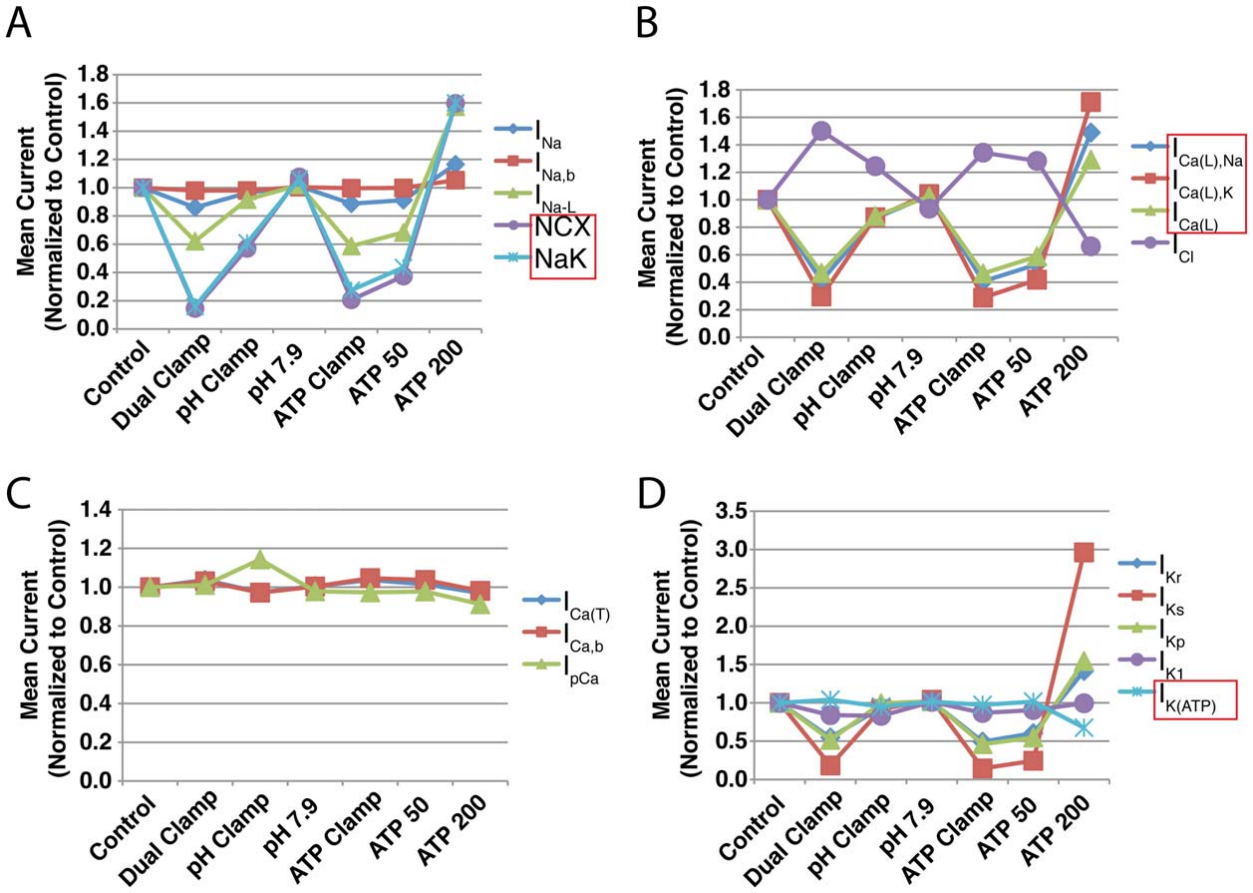
Normalized mean currents throughout 10 minutes of reperfusion. For each of 17 transmembrane currents, mean current in each simulation summarized in Fig. 3 is normalized to the corresponding control. Currents that are directly modulated by pH and/or phosphometabolite concentrations are shown in red boxes. Simulations were run using a pacing rate of 3 Hz. Mean currents for the full set of 3 Hz simulations are provided in Text S1.

For each simulation, we calculated the mean current through all 17 electrogenic transmembrane flux sources over the 10 minutes of simulated reperfusion. These values are reported in tables in Text S1. In each table, mean currents for the control and dual clamp (both 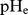 and phosphometabolites clamped at end-ischemic values) simulations are also provided. These mean currents were normalized to their corresponding control values and are illustrated in Fig. 7 for the simulations shown in Fig. 3 (3 Hz only).

Unsurprisingly, both NCX and NaK experienced substantial reductions in current when 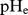 recovery was inhibited (Fig. 7A (purple and light blue)). Both of these exchangers saw an approximate 40 percent reduction in mean current when 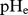 was clamped, and demonstrated a direct relationship between mean current and the amount of pH recovery allowed. Currents through the L-type calcium channel (Fig. 7B (blue, red, and green)) and, to a lesser extent, 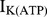 (Fig. 7D (light blue)) manifested changes when different degrees of pH recovery were allowed. For 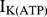, these effects result from differences in ADP concentration (this current is sensitive to changes in ADP, which in turn responds to changes in pH), but for 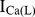, which is not modulated by ADP in our model, these effects can only be the result of changes in ion concentrations and hence driving force. Additional currents, not subject to direct modulation by either pH or phosphometabolites, exhibited substantial alterations in mean current: 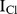 (Fig. 7B (purple)), 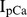 (Fig. 7C (green)), 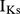 (Fig. 7D (red)), and 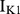 (Fig. 7D (purple)). Individual current traces for NCX, NaK, and 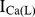 are shown below their corresponding action potentials in Fig. 5.

When phosphometabolite availability was altered during reperfusion, some of the most dramatic results were observed in components sensitive to phosphometabolite concentrations. NaK (Fig. 7A (purple)) and L-type calcium channel currents (Fig. 7B (blue, red, and green lines)) experienced greater than 80 percent reductions in mean current at the lowest ATP and PCr concentrations. However, other currents exhibited significant changes despite not being modulated by phosphometabolites. Notably, NCX current changes were nearly identical to those exhibited by NaK (Fig. 7A (purple and light blue, respectively)). In addition, 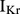, 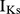 and 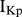 exhibited marked reductions in mean current (Fig. 7D (dark blue, red and green lines, respectively)) at low concentrations of ATP and PCr. Conversely, these currents, especially 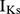, increased above control values when ATP and PCr concentrations were increased to levels greater than the control scenario. 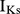 was dramatically greater during reperfusion in the ATP 200 simulation (see Fig. 5, bottom row). This appears to be due to the fact that the equation determining reversal potential for this current (adopted from [15]) includes a term for intracellular sodium concentration, which was significantly lower in these simulations.

In summary, the largest alterations to transmembrane current were seen in NCX, NaK, 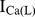, and 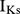. Additionally, the effects were more severe when phosphometabolite status was perturbed during reperfusion than when pH recovery was perturbed.

### Ion Concentrations and pH

**Figure 8:**
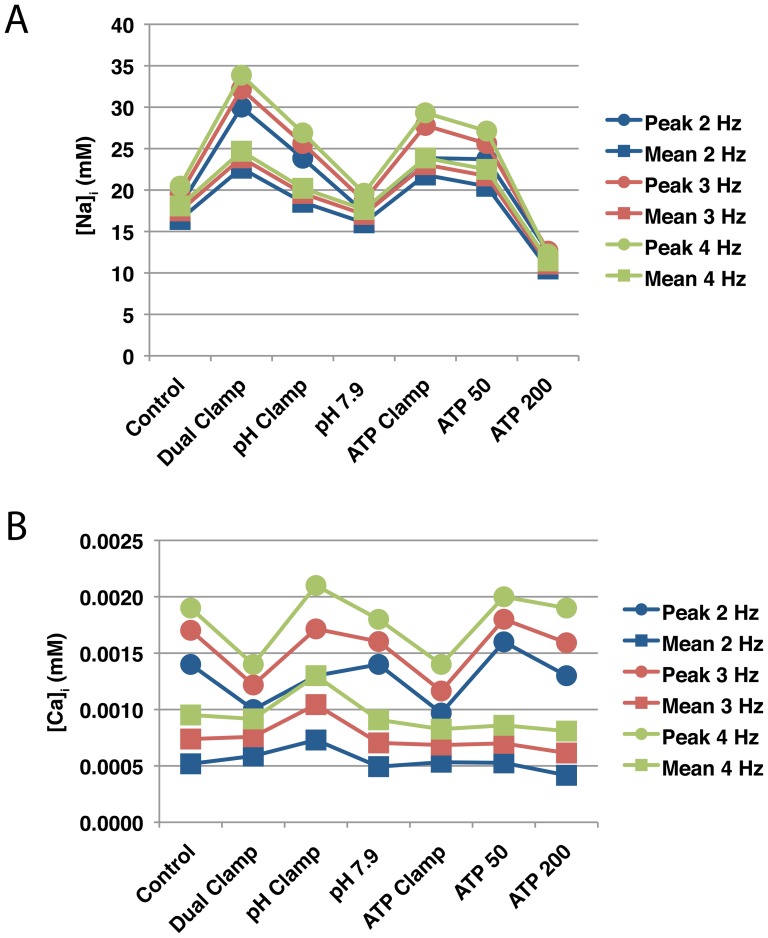
Sodium and calcium concentrations during simulated reperfusion. Each of the seven simulation protocols summarized in Fig. 3 were run using three pacing rates: 2 Hz (blue), 3 Hz (red), and 4 Hz (green). For each simulation, the peak (circles) and mean (squares) concentrations of intracellular sodium (A) and calcium (B) were recorded during reperfusion. Results for the full compliment of 3 Hz simulations can be found in Text S1.

When pH recovery was inhibited or phosphometabolite concentrations were not allowed to return to their control values during reperfusion, intracellular sodium concentrations were elevated relative to control (Fig. 8A). Conversely, sodium concentrations were lower than control when either pH recovered to a more basic value or higher concentrations of ATP and PCr were allowed. Reducing phosphometabolite availability tended to produce higher peak concentrations than impairing pH recovery. With regard to mean sodium load, phosphometabolite reductions appear to play a more significant role than pH — the differences between the dual clamp simulation and pH and ATP clamp simulations were 4.30 and 0.82 mM, respectively.

Inhibiting pH recovery or ATP and PCr during reperfusion produced similar peak intracellular calcium loads, although these maximum loads occurred not when any of the parameters were clamped at their end-ischemic values (i.e. pH and ATP Clamp simulations), but rather when a partial recovery of pH was allowed or the end-reperfusion target for ATP and PCr was somewhere between the end-ischemic clamp and the control protocol (Fig. 8B (circles) and see Text S1). In terms of mean calcium load, the highest concentration was observed when extracellular pH was clamped at its end-ischemic value (Fig. 8B (squares)). Allowing extracellular pH to recover to a more basic value, or allowing more ATP and PCr during reperfusion, resulted in lower calcium concentrations relative to control. The dual clamp simulation also produced lower peak calcium load than control, but mean calcium load was slightly higher than control and SR calcium was relatively depleted (not shown). This loss of dynamic range is not surprising, as calcium-handling components of the model are inhibited by low pH and ATP availability.

The relationships between perturbations to pH and phosphometabolite status and sodium and calcium loads persisted across the three pacing rates that we investigated. As can be seen in Fig. 8A, the highest sodium loads were observed in the Dual and ATP Clamp simulations at all pacing rates. Similarly, the lowest sodium loads at all three rates were observed in the ATP 200 simulations. The same was true for intracellular calcium load (Fig. 8B); general trends across the seven simulation protocols persisted regardless of the pacing rate used.

**Figure 9:**
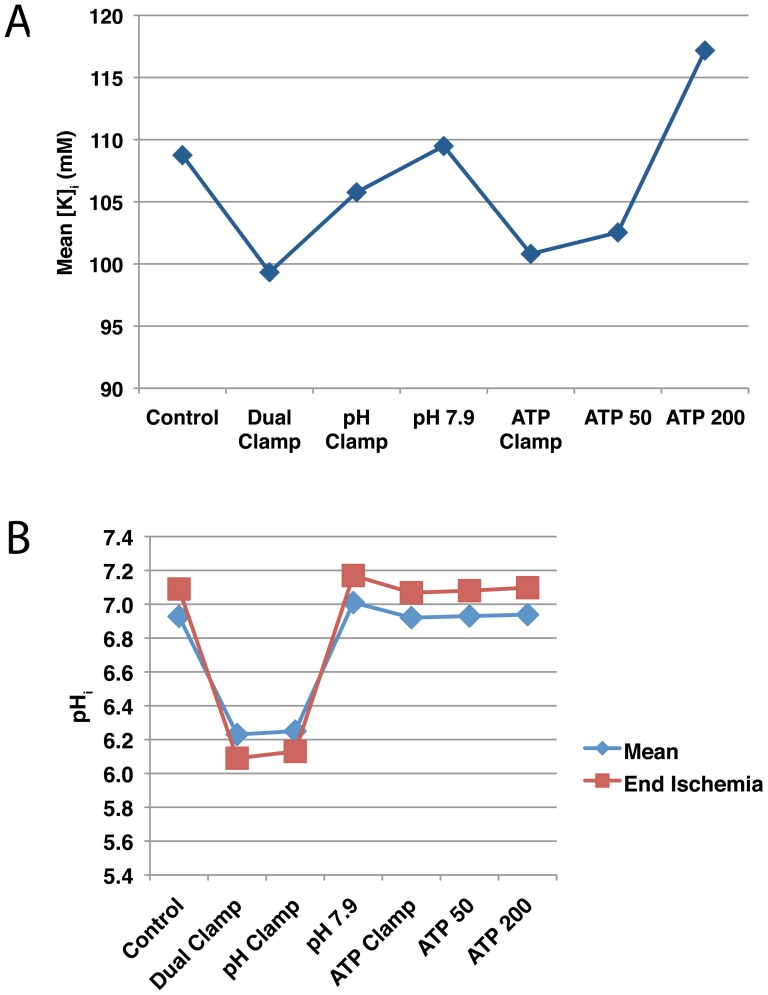
Intracellular pH and potassium concentrations during simulated reperfusion. (A) Mean intracellular potassium concentrations. (B) Mean intracellular pH (blue diamonds) and pH after 10 minutes of reperfusion (red squares). These simulations (summarized in Fig. 3) were run using a pacing rate of 3 Hz. Full results can be found in Text S1.

Inhibiting extracellular pH recovery or phosphometabolite availability reduced mean intracellular potassium load throughout reperfusion (Fig. 9A), though impaired phosphometabolite recovery produced a more substantial effect.

**Figure 10:**
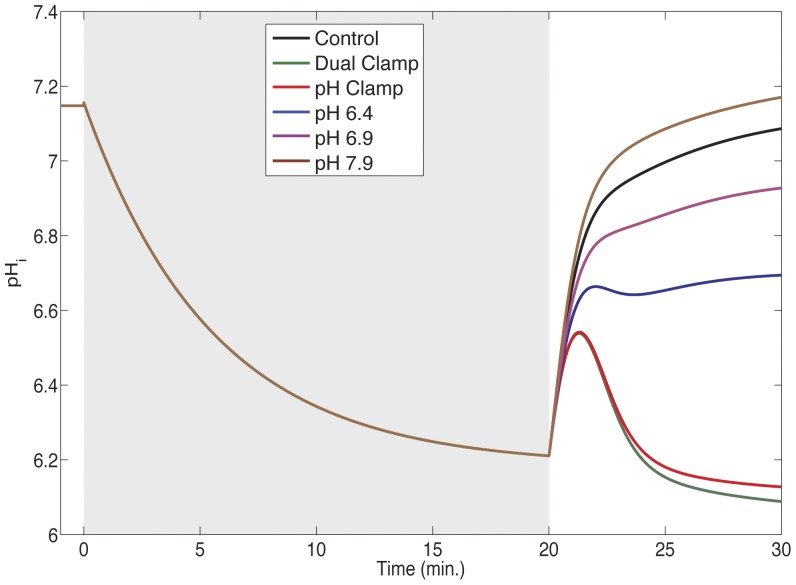
Intracellular pH profiles in simulations with variable extracellular pH recovery. Intracellular pH values during simulated ischemia (gray region) and reperfusion are shown for simulations in which extracellular pH was clamped at the end-ischemic value (green and red lines) or allowed to recover to a specified target (blue, purple, and brown lines). Extracellular pH profiles for these simulations can be seen in Text S1.

As expected, allowing higher end-reperfusion extracellular pH targets was associated with higher end-reperfusion and mean intracellular pH values (Fig. 9B and Text S1). On the other hand, inhibiting phosphometabolite availability resulted in trivial changes to intracellular pH recovery during reperfusion. The lowest mean and end intracellular pH values were observed in the dual clamp and pH clamp simulations, with the dual clamp scenario resulting in slightly lower pH values. Also, these were the only two simulations in which the end pH (Fig. 9B (red squares)) was lower than the mean pH (blue diamonds). This appears to be related to the fact that even when extracellular pH is clamped at its end-ischemic value, there is still a significant, albeit temporary recovery in intracellular pH during the first two or so minutes of reperfusion. Following this recovery, 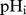 abruptly decreases, falling to levels slightly lower than observed at the beginning of reperfusion (Fig. 10 (red and green lines)). This transient recovery is likely created by a washout of extracellular carbon dioxide, allowing a rapid decrease in intracellular carbon dioxide levels. There may also be a smaller contribution from increased bicarbonate flux through the NBC as extracellular bicarbonate is replenished during reperfusion.

Mean ADP concentrations during reperfusion are reported in Text S1. Of note is the mean concentration in the phosphometabolite clamp simulation (“ATP Clamp”). This extraordinarily high concentration is caused by the fact that intracellular pH is allowed to recover (as opposed to the dual clamp simulation) but ATP and PCr are held at their low end-ischemic concentrations. The relationship between ATP, PCr, intracellular pH, and ADP can be seen in Text S1 (Eq. S6). When intracellular pH is allowed to recover, relatively low proton concentrations, together with low PCr concentration (which is substantially greater than ATP concentration and thus plays a bigger role), result in a high concentration of ADP.

In summary, suppressing either pH recovery or the recovery of ATP and PCr during reperfusion was associated with elevated concentrations of intracellular sodium and calcium, though the correlation was weaker for the latter. Making more ATP and PCr available than in the control simulation was associated with reduced sodium and calcium loads.

## Discussion

### Currents Affecting Action Potential Morphology

As Figs. 4 and 5 illustrate, reducing ATP and PCr availability during reperfusion reduces the ability of action potentials to recover toward their normal morphology to a greater extent than does inhibiting pH recovery. When ATP and PCr were clamped at their end-ischemic concentrations during reperfusion, the mean APD was reduced by almost 29 ms relative to control, whereas a less than 3 ms difference in mean APD was noted when extracellular pH was clamped (see values provided in Text S1). In addition, mean APD was reduced even further, by an additional 15.4 ms, in the “ATP 25” simulation, where additional PCr recovery was allowed but ATP availability was further suppressed. Following a similar trend, mean APA during reperfusion was reduced by greater than 10 ms in the phosphometabolite clamp simulation, compared to less than 3 ms in the pH clamp simulation.

Conversely, increasing ATP and PCr availability during reperfusion was much more beneficial, in terms of recovering toward preischemic APD and APA, than increasing the end-reperfusion target of 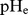 to 7.9. Increasing the end-reperfusion targets of [ATP] and [PCr] to 200 percent that of control allowed APD and APA to recover to values greater than observed in the control simulation.

Shorter action potential durations and amplitudes can stem from reductions to depolarizing currents or increases in repolarizing currents. Of the currents in the model that are directly modulated by phosphometabolites, the only one that experienced a change in flux consistent with APD and APA shortening is 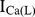, which passes calcium (and to a lesser extent, sodium) into the cell, as well as potassium out of the cell. Mean 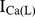 and 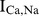 currents fell to approximately 20 percent of their control values when ATP and PCr were clamped, and fell even further when ATP and PCr targeted 25 percent of their control concentrations during reperfusion (Fig. 7B and Text S1). However, reduced pH during simulations also impaired flux through these channels. During the pH clamp simulation, mean 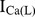 and 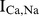 were reduced by just under 20 percent relative to control due to elevated calcium decreasing inward driving force and enhancing calcium-dependent inactivation.

The other two currents in the model directly modulated by phosphometabolites, 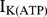 and NaK, are both repolarizing currents. As both of these sources exhibited reduced flux during pH and phosphometabolite inhibition, their currents cannot directly account for impaired APD and APA recovery in these simulations, although reduced NaK flux contributes to increased sodium load, which plays a role in impaired recovery.

However, several other channels in the model exhibited reduced depolarizing current. The first is NCX, which is modulated by intracellular pH and is sensitive to intracellular sodium (which in turn is strongly affected by NaK, a pump modulated by ATP, ADP, and PCr). NCX experienced an approximately 40 percent reduction in mean current in the pH clamp simulation (Fig. 7A), and a much greater reduction of approximately 80 percent in the phosphometabolite clamp simulation. These reductions in current were caused by acidic inhibition and elevated sodium loads (which were more severe in the phosphometabolite clamp simulations, see Fig. 8A), producing a shift from forward mode action toward reverse mode (compare red to green dashed lines in NCX current traces in Fig. 5).

In addition, 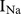 and 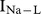 experienced reductions in mean current of 12 and 41 percent, respectively, relative to control in the phosphometabolite clamp simulation (Fig. 7A and Text S1), compared to 4 and 8 percent reductions in the pH clamp simulation. Some simulations also revealed a small reduction of 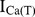 (Fig. 7C (blue line) and increased 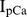 (Fig. 7C (green line)), both of which contribute to APD shortening.

**Figure 11:**
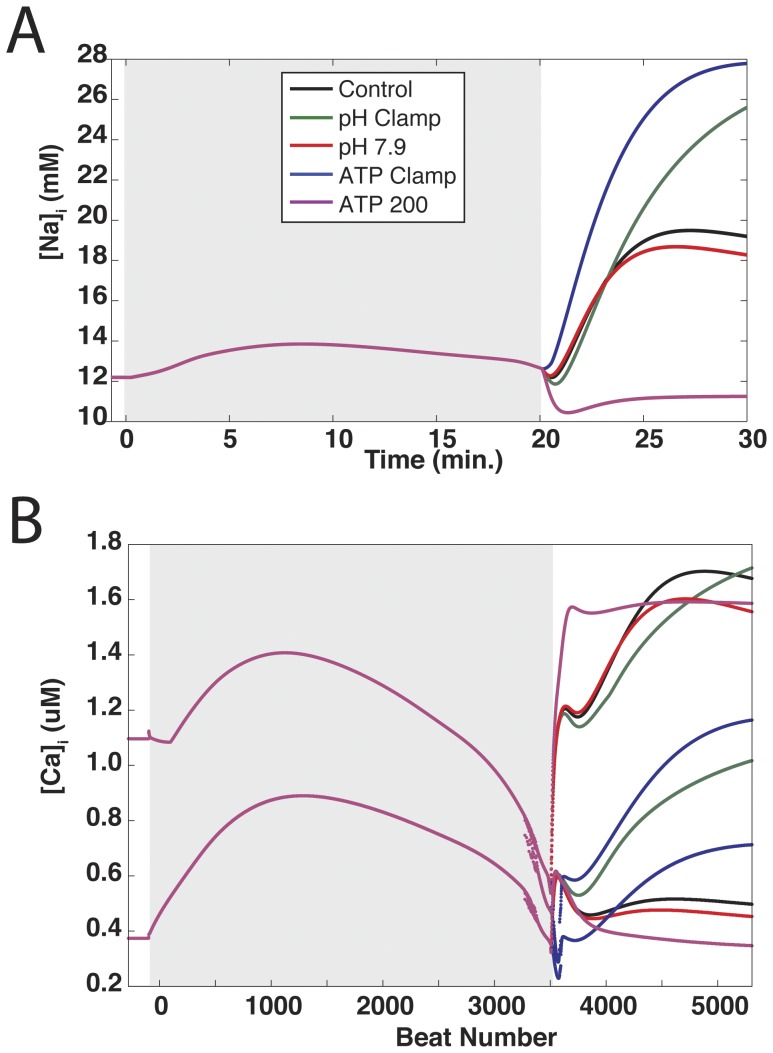
Intracellular sodium and calcium profiles from select simulations. Intracellular sodium (A) and maximum and minimum intracellular calcium (B) concentrations are plotted during ischemia (gray regions) and reperfusion for five simulations: control (black), pH clamp (green), pH recovery to 7.9 (red), phosphometabolite clamp (blue), and phosphometabolite recovery to 200 percent of control (purple).

As noted in the Results section, there is an apparent overshoot phenomenon in many simulations wherein APD and APA values are at their greatest during the first one to two minutes of reperfusion, then settle at lower values at the end of the reperfusion period. This appears to be due to the fact that processes that occur during reperfusion occur on different time scales. In our model, ATP and PCr concentrations recover to their target values relatively quickly, over the first 1–2 minutes of reperfusion. This will affect sodium homeostasis through a rapid increase in NaK pump function, producing a transient decrease in sodium load that scales with the amount of ATP and PCr recovery that is allowed. In Fig. 11A, intracellular sodium concentrations throughout five simulations are shown. Within the first 1–2 minutes of reperfusion, most of these simulations exhibit a transient “dip” in sodium load before sodium begins to accumulate again. When ATP and PCr are clamped throughout reperfusion (blue line), there is practically no dip. On the opposite end of the spectrum, allowing ATP and PCr to recover to twice their control targets (purple line) transforms the dip into a more substantial decrease of sodium load (approximately 2 mM). On the other hand, extracellular pH and ion concentrations require several more minutes to recover (10 minutes for pH). As extracellular pH recovers, sodium influx via NHE will resume. Conversely, sodium efflux via NaK will be reduced as extracellular potassium concentrations return to normal over the first several minutes of reperfusion. Together, these produce a notable increase in sodium load throughout the latter phase of reperfusion.

Fig. 11B illustrates maximum and minimum intracellular calcium concentrations for the same five simulations. A clear two-stage accumulation of calcium can be seen in these cases — a peak within the first 1–2 minutes of reperfusion, followed by a slight decrease, then a more profound subsequent increase in calcium load. This is not unexpected, as many of the calcium-handling components in the model are sensitive to both pH and phosphometabolites, which as discussed above, recover on different time scales. In addition, calcium homeostasis is tightly linked to sodium homeostasis (via NCX), which exhibits two different phases of recovery as discussed above.

Of course the question is, how do sodium and calcium homeostasis relate to action potential morphology? With respect to intracellular sodium, higher concentrations appear to be correlated with shorter APD and APA, which would be expected as higher sodium loads reduce inward driving force for depolarizing sodium currents. The situation for calcium is different, as there is no clear correlation between mean calcium load and mean APD or APA. Examining Fig. 11B reveals some correlation between calcium transient amplitude and APD/APA. For example, during reperfusion in the ATP 200 simulation, in which the largest APD and APA values were noted (along with no fall off of APD or APA after the initial rise) (Fig. 11B (purple lines)), maximum calcium concentrations rise quickly, dip slightly, and then stay at about the same level. Minimum concentrations stabilize after a couple of minutes and change relatively little afterward. On the other hand, in the ATP clamp simulation (blue lines), where APD (and to a lesser extent, APA) were significantly reduced and fall off substantially in the latter minutes of reperfusion, the differences between maximum and minimum calcium loads are smaller. Possibly more importantly, calcium concentrations continue to substantially change during the latter phase of reperfusion. In short, the progressive deterioration of APD and APA during reperfusion appears to be a function of both intracellular sodium load and calcium dynamics.

There is a strong correlation between RMP (Fig. 4C) and intracellular potassium concentration (Fig. 9A). In turn, there are strong correlations between intracellular potassium concentration and phosphometabolite availability, as well as potassium and pH status during reperfusion. These correlations are expected, as the NaK, which relies upon ATP and PCr availability for proper function and is inhibited by low pH, is the sole pathway in the model through which potassium can reenter the cell. Since extracellular potassium recovery is identical in all simulations, differences in RMP between simulations would be expected to be dominated by differences in intracellular potassium.

### Relationships between pH, Phosphometabolites, and Ion Concentrations

Of the currents considered here that are directly modulated by pH and phosphometabolite status, NaK and NCX play the largest roles in sodium balance. NaK, which removes sodium from the cell, is inhibited by acidosis, reduced ATP and PCr availability, and increased concentrations of ADP. In the series of simulations examining differential pH recovery, there is a relationship between intracellular pH (Fig. 9B and Text S1) and intracellular sodium load (Fig. 8A and Text S1)—lower pH values are correlated with higher sodium loads. In the series of simulations examining differential ATP and PCr availability, there is a correlation between sodium load and the concentration of phosphometabolites during reperfusion. Thus, the relationships between sodium balance, pH and phosphometabolites in the model are relatively straightforward.

As mentioned previously, the NCX operates in two modes: a forward mode in which sodium moves into the cell and calcium leaves, and a reverse mode in which calcium enters and sodium leaves. At higher intracellular sodium loads, the NCX will spend more time in the reverse mode. Still, even when spending more time in reverse mode, NCX net flux is still in the forward direction. This means that inhibiting the NCX, as occurs under acidic conditions, will have the benefit of attenuating sodium overload, but will exacerbate calcium overload.

Two other components that affect sodium homeostasis, but were not included in the current study because they are not electrogenic, are NHE and NBC. As discussed in [25], these exchangers play a relatively minor role under normal conditions, but contribute significantly greater sodium influx under acidic conditions (roughly 15 and 12 percent of total sodium influx at pH 6.9, respectively).

The relationships governing intracellular calcium load are much more complex and involve processes that are beyond the scope of this study. As has been discussed already, calcium homeostasis is strongly coupled to sodium homeostasis, giving it indirect links to phosphometabolite status and pH inhibition via NaK. Calcium homeostasis is also directly linked to pH via NCX inhibition and ATP availability via 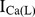. In addition, calcium concentrations are affected by several other processes, including uptake into the SR via SERCA (which responds to changes in pH, ATP, and ADP concentrations), SR release via RyR (sensitive to pH, and myoplasmic calcium), and 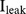 (which depends on SR calcium load).

Ion channel kinetics, action potential morphology, and ion homeostasis are all coupled and exhibit rate dependence. As such, it was important to assess whether our conclusions were dependent upon pacing rate. As Figs. 6 and 8 illustrate, while there are differences across the three pacing rates we employed, the general trends remain consistent. Regardless of rate, phosphometabolite status plays a larger role in dictating action potential morphology and sodium and calcium load during reperfusion than does pH.

### Limitations

The primary aim of this study was to investigate the relative influences of pH and phosphometabolite availability on processes that lead to detrimental changes in action potential morphology that are associated with the development of reentry. While changes in APD, APA, and RMP can be observed, unidirectional conduction block and reentry cannot, as this is a single-cell model. In addition, cellular coupling and electrotonic effects play a role in making tissue more prone to reentry [26]. These effects cannot be taken into account in these single-cell simulations.

In this study, we manipulated components of the extracellular pH system directly, forcing the return of the variables representing extracellular pH, bicarbonate and carbon dioxide to preischemic levels. This approach was taken in order to simulate the washout of acidic extracellular fluid, which is replaced with fluid that contains normal concentrations of bicarbonate and carbon dioxide. However, this removes a certain amount of feedback between pH, carbon dioxide, and bicarbonate during simulations in which we inhibited extracellular pH recovery. In simulations where partial pH recovery was allowed, we set a target for extracellular pH but forced the return of bicarbonate and carbon dioxide to normal levels. This is not altogether unrealistic: in experiments where intracellular pH recovery has been inhibited by reperfusion with an acidic buffer [22], carbon dioxide concentrations were kept at their normal levels. The pH of the reperfusion buffer in this type of experiment is commonly reduced by decreasing bicarbonate concentration [22] or adding hydrochloric acid [23], which is not what we did in our study. This may have an effect on the amount of intracellular pH recovery and sodium influx, with our approach likely producing increased bicarbonate and sodium influx via the NBC. However, our goal was to examine the effects of differential pH recovery on the inside of the cell, which we were able to produce. In previous simulations examining combinations of NHE and NBC inhibition (results not shown), we noted that reducing NBC flux by 50 percent in addition to NHE blockade cause the final intracellular pH to decrease by less than 0.1 unit, compared to NHE blockade alone.

Also, as is apparent from the preceding discussion, in addition to direct modulation by metabolites and pH, ion channel currents are modulated by changes in ion concentration and membrane voltage. For example, 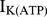 is modified by ATP and ADP concentrations. Furthermore, 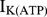 also varies with differences in potassium concentrations, as well as the resting membrane potential, both of which affect the driving force. In turn, potassium homeostasis, which affects resting membrane potential and action potential duration, is influenced by NaK (which is modulated by ATP and ADP). Thus, quantifying the consequences of changing ATP and ADP availability on 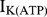 flux are confounded by indirect effects brought about by other components in the system. These confounding effects prevent this study from providing a specific analysis of the direct consequences of modulating phosphometabolites and pH on individual components. Such an analysis was not the goal of this study, although the model can be used to execute such a study.

## Conclusions

The study presented here suggests that the pathological changes in phosphometabolite concentrations that occur during ischemia are more consequential than changes in pH when considering action potential morphology. Preventing intracellular pH recovery during simulated reperfusion had relatively modest effects on action potential duration, amplitude, and resting membrane potential. In contrast, preventing recovery of phosphometabolites resulted in dramatic reductions in action potential duration and amplitude compared to control, and resulted in more membrane depolarization.

With respect to intracellular sodium and calcium, the results were not as straightforward. Inhibiting phosphometabolite recovery alone produced more extreme sodium overloads during reperfusion than inhibiting pH recovery alone, although inhibiting both produced the highest sodium loads of all. On the other hand, allowing some pH or phosphometabolite recovery produced the highest peak calcium loads, although the highest mean calcium load was observed when no pH recovery was allowed. These findings highlight the complex relationships between pH, ATP and ADP concentrations, and ion concentrations which are mediated by numerous cell components.

We were interested in examining the differential roles of pH and phosphometabolite recovery during reperfusion because these are all targets that can be manipulated *in vivo* and/or the clinical setting. The results of these simulations, along with the results of our previous study [25], suggest that efforts directed at increasing ATP and/or PCr availability during reperfusion may be more useful than manipulating pH during reperfusion, particularly in terms of restoring preischemic action potential morphology as well as reducing sodium and calcium overload.

Model code is available from the authors upon request.

## Supporting Information

Text S1 **Numerical results and figures for the full set of 12 simulations performed at a pacing rate of 3 Hz, as well as a list of equations pertinent to phosphometabolite representation in the model.**
(PDF)
