# Supplementary material for: The Relative Influences of Phosphometabolites and pH on Action Potential Morphology during Myocardial Reperfusion: A Simulation Study
Source: PLoS One. 2012 Nov 7;7(11):e47117. doi: 10.1371/journal.pone.0047117 (PMC3492384; doi:10.1371/journal.pone.0047117)
Supplement: Text S1 — Numerical results and figures for the full set of 12 simulations performed at a pacing rate of 3 Hz, as well as a list of equations pertinent to phosphometabolite representation in the model. (PDF) [file pone.0047117.s001.pdf]

Text S1

Supplementary Material for “The Relative Influences of Phosphometabolites and pH on Action Potential Morphology During Myocardial Reperfusion: A Simulation Study.”

Byron N. Roberts and David J. Christini

Table S1: Action Potential Duration (90) During Initial 10 Minutes of Reperfusion (ms)

	Pre	R0	R1	R2	R3	R4	R5	R6	R7	R8	R9	R10	Mean
Control	109.0	16.5	88.5	92.0	88.5	84.5	82.0	81.0	80.0	79.5	79.5	79.5	83.5
Dual Clamp	109.0	16.5	66.5	67.5	64.5	61.5	59.5	57.5	56.0	54.5	53.5	52.5	59.4
pH Clamp	109.0	16.5	87.5	90.0	86.5	83.0	80.5	79.0	77.0	75.5	74.5	73.5	80.7
pH 6.4	109.0	16.5	88.0	91.5	88.0	83.5	80.0	78.0	76.0	74.5	73.5	72.5	80.6
pH 6.9	109.0	16.5	88.5	92.0	88.0	84.0	81.0	79.5	78.0	77.0	76.5	76.5	82.1
pH 7.9	109.0	16.5	88.5	92.0	88.5	85.0	82.5	82.0	81.5	81.5	81.5	81.5	84.4
ATP Clamp	109.0	16.5	66.5	66.5	61.5	57.0	53.5	51.5	49.5	48.5	47.5	47.0	54.9
ATP 25	109.0	16.5	48.0	48.5	45.0	41.5	38.5	37.0	35.5	34.5	33.5	33.0	39.5
ATP 50	109.0	16.5	72.5	73.0	68.0	63.0	59.0	56.0	54.5	53.0	52.0	51.5	60.2
ATP 75	109.0	16.5	83.0	85.0	80.0	75.5	72.0	70.0	68.0	67.0	66.5	66.5	73.4
ATP 150	109.0	16.5	95.5	102.0	101.0	99.5	99.0	99.0	99.0	99.0	99.0	99.0	99.2
ATP 200	109.0	16.5	101.0	109.5	111.0	111.0	110.5	111.0	111.0	111.0	110.5	110.5	109.7

Table S2: Action Potential Amplitude During Initial 10 Minutes of Reperfusion (mV)

	Pre	R0	R1	R2	R3	R4	R5	R6	R7	R8	R9	R10	Mean
Control	118.4	36.1	101.9	110.1	110.9	109.7	109.5	109.1	108.9	108.9	108.9	108.9	108.7
Dual Clamp	118.4	36.1	90.2	105.1	105.2	102.2	99.1	97.1	94.8	92.6	90.5	88.6	96.5
pH Clamp	118.4	36.1	101.8	111.2	111.6	109.2	106.8	105.7	104.7	103.9	103.2	102.6	106.0
pH 6.4	118.4	36.1	101.9	110.3	110.6	108.5	107.8	106.7	106.3	105.8	105.4	105.2	106.9
pH 6.9	118.4	36.1	101.9	110.0	110.7	109.1	108.7	108.1	107.7	107.5	107.4	107.3	107.8
pH 7.9	118.4	36.1	101.9	110.2	111.1	110.5	110.1	109.9	109.8	109.8	109.8	109.9	109.3
ATP Clamp	118.4	36.1	89.9	103.5	103.7	101.8	99.8	98.9	97.7	96.5	95.4	94.6	98.2
ATP 25	118.4	36.1	78.9	103.7	104.5	102.2	100.6	99.5	98.1	96.7	95.4	95.0	97.5
ATP 50	118.4	36.1	94.7	105.0	105.6	103.6	101.7	101.0	100.0	99.1	98.3	97.5	100.6
ATP 75	118.4	36.1	99.5	107.2	108.0	106.4	105.7	105.0	104.6	104.3	104.2	104.1	104.9
ATP 150	118.4	36.1	104.5	113.8	117.0	116.6	116.2	116.1	116.3	116.3	116.1	115.9	114.9
ATP 200	118.4	36.1	106.2	119.1	122.0	122.1	121.8	121.8	121.9	121.8	121.5	121.2	119.9

Table S3: Resting Membrane Potential During Initial 10 Minutes of Reperfusion (mV)

	Pre	R0	R1	R2	R3	R4	R5	R6	R7	R8	R9	R10	Mean
Control	-78.0	-48.4	-69.2	-75.9	-77.3	-77.4	-77.4	-77.2	-77.1	-77.0	-76.9	-76.8	-76.2
Dual Clamp	-78.0	-48.4	-68.5	-75.2	-76.4	-76.1	-75.5	-75.0	-74.4	-73.9	-73.4	-73.0	-74.1
pH Clamp	-78.0	-48.4	-69.3	-76.2	-77.5	-77.5	-77.1	-76.8	-76.4	-76.1	-75.8	-75.5	-75.8
pH 6.4	-78.0	-48.4	-69.3	-75.9	-77.2	-77.2	-77.0	-76.8	-76.6	-76.4	-76.2	-76.1	-75.9
pH 6.9	-78.0	-48.4	-69.2	-75.9	-77.2	-77.3	-77.2	-77.0	-76.8	-76.7	-76.6	-76.4	-76.0
pH 7.9	-78.0	-48.4	-69.2	-75.9	-77.4	-77.6	-77.5	-77.4	-77.3	-77.2	-77.2	-77.1	-76.4
ATP Clamp	-78.0	-48.4	-68.3	-75.0	-76.3	-76.2	-75.9	-75.6	-75.2	-75.0	-74.7	-74.5	-74.7
ATP 25	-78.0	-48.4	-68.2	-74.9	-76.2	-76.1	-75.7	-75.4	-75.1	-74.8	-74.5	-74.3	-74.5
ATP 50	-78.0	-48.4	-68.5	-75.2	-76.5	-76.5	-76.2	-75.9	-75.7	-75.4	-75.2	-75.0	-75.0
ATP 75	-78.0	-48.4	-68.9	-75.5	-76.9	-76.9	-76.8	-76.6	-76.4	-76.2	-76.1	-75.9	-75.6
ATP 150	-78.0	-48.4	-69.7	-76.7	-78.2	-78.5	-78.5	-78.4	-78.4	-78.3	-78.3	-78.2	-77.3
ATP 200	-78.0	-48.4	-70.1	-77.3	-79.0	-79.3	-79.3	-79.2	-79.2	-79.1	-79.1	-79.0	-78.1

Table S4: Mean Currents (uA/uF) During Reperfusion (pH Simulations)

	Control	Dual Clamp	pH Clamp	pH 6.4	pH 6.9	pH 7.9
I_{Na}	-0.2022	-0.1739	-0.1948	-0.1958	-0.1992	-0.2044
$I_{Na,b}$	-0.2269	-0.2223	-0.2225	-0.2237	-0.2255	-0.2278
$I_{Na\text{-}L}$	-0.0119	-0.0074	-0.0109	-0.0111	-0.0116	-0.0121
NCX	-0.3058	-0.0445	-0.1745	-0.2246	-0.2704	-0.3292
NaK	0.4911	0.0783	0.3000	0.3838	0.4433	0.5215
$I_{Ca(L),Na}$	-0.1218	-0.0500	-0.1056	-0.1095	-0.1163	-0.1256
$I_{Ca(L),K}$	0.0613	0.0182	0.0535	0.0546	0.0580	0.0639
$I_{Ca(L)}$	-1.0362	-0.4851	-0.9138	-0.9514	-0.9991	-1.0609
I_{Cl}	-0.3560	-0.5343	-0.4431	-0.4300	-0.3890	-0.3330
I_{Kr}	0.1445	0.0793	0.1356	0.1369	0.1411	0.1470
I_{Ks}	0.1119	0.0205	0.1027	0.1004	0.1063	0.1164
I_{Kp}	0.0604	0.0313	0.0600	0.0576	0.0589	0.0617
I_{K1}	0.8181	0.6872	0.6794	0.7634	0.7983	0.8287
$I_{Ca(T)}$	-0.0740	-0.0767	-0.0720	-0.0726	-0.0733	-0.0744
$I_{Ca,b}$	–0.4852	-0.5001	-0.4717	-0.4791	-0.4826	-0.4869
I_{pCa}	0.9589	0.9700	1.0960	1.0328	0.9904	0.9385
$I_{K(ATP)}$	0.3115	0.3243	0.2961	0.2976	0.3051	0.3161

Table S5: Mean Currents (uA/uF) During Reperfusion (Phosphometabolite Simulations)

	Control	Dual Clamp	ATP Clamp	ATP 25	ATP 50	ATP 75	ATP 150	ATP 200
I_{Na}	-0.2022	0.1739	-0.1793	-0.1811	-0.1844	-0.1922	-0.2217	-0.2357
$I_{Na,b}$	-0.2269	-0.2223	-0.2261	-0.2373	-0.2266	-0.2249	-0.2334	-0.2391
$I_{Na\text{-}L}$	-0.0119	-0.0074	-0.0070	-0.0050	-0.0081	-0.0101	-0.0156	-0.0187
NCX	-0.3058	-0.0445	-0.0632	-0.0089	-0.1145	-0.2140	-0.4355	-0.4883
NaK	0.4911	0.0783	0.1352	0.0920	0.2140	0.3527	0.6941	0.7856
$I_{Ca(L),Na}$	0.1218	-0.0500	-0.0495	-0.0187	-0.0646	-0.0962	-0.1620	-0.1814
$I_{Ca(L),K}$	0.0613	0.0182	0.0176	0.0052	0.0257	0.0444	0.0912	0.1050
$I_{Ca(L)}$	-1.0362	-0.4851	-0.4805	-0.1892	-0.6096	-0.8626	-1.2527	-1.3384
I_{Cl}	-0.3560	-0.5343	-0.4781	-0.5419	-0.4568	-0.4012	-0.2812	-0.2355
I_{Kr}	0.1445	0.0793	0.0713	0.0370	0.0874	0.1200	0.1808	0.2046
I_{Ks}	0.0723	0.0132	0.0162	0.0017	0.0274	0.0658	0.2303	0.3315
I_{Kp}	0.0604	0.0313	0.0278	0.0130	0.0331	0.0477	0.0818	0.0934
I_{K1}	0.8181	0.6872	0.7108	0.7823	0.7414	0.7738	0.8412	0.8136
$I_{Ca(T)}$	-0.0740	-0.0767	-0.0767	-0.0703	-0.0751	-0.0742	-0.0733	-0.0717
$I_{Ca,b}$	-0.4852	-0.5001	-0.5079	-0.5366	-0.5034	-0.4905	-0.4796	-0.4760
I_{pCa}	0.9589	0.9700	0.9332	0.7655	0.9378	0.9747	0.9083	0.8749
$I_{K(ATP)}$	0.3115	0.3243	0.3034	0.2372	0.3164	0.3248	0.2648	0.2106

Table S6: pH_i and Ion Concentrations (mM) During Reperfusion (pH Simulations)

	Control	Dual Clamp	pH Clamp	pH 6.4	pH 6.9	pH 7.9
Peak $[Na^+{}_i]$	19.50	32.17	25.61	23.49	20.91	18.69
Mean $[Na^+{}_i]$	17.48	23.89	19.59	19.17	18.17	17.06
Peak $[Ca^{2+}{}_i]$	0.001702	0.001218	0.001715	0.001974	0.001842	0.001603
Mean $[Ca^{2+}{}_i]$	0.000738	0.000758	0.001044	0.000886	0.000796	0.000704
Mean $[Ca_{SR}]$	3.081	0.701	2.827	3.022	3.059	3.083
Mean CaiT Ampl	0.001000	0.000282	0.000635	0.000938	0.000998	0.000981
Mean $[K^+{}_i]$	108.75	99.33	105.76	106.79	107.78	109.49
End pH_i	7.09	6.09	6.13	6.69	6.93	7.17
Mean pH_i	6.93	6.23	6.25	6.64	6.81	7.01
Mean [ADP]	0.0197	0.1448	0.0042	0.0097	0.0146	0.0237

Table S7: pH_i and Ion Concentrations (mM) During Reperfusion (Phosphometabolite Simulations)

	Control	Dual Clamp	ATP Clamp	ATP 25	ATP 50	ATP 75	ATP 150	ATP 200
Peak $[Na^+{}_i]$	19.50	32.17	27.79	25.93	25.61	22.91	14.11	12.63
Mean $[Na^+{}_i]$	17.48	23.89	23.07	21.60	21.70	19.91	13.41	11.07
Peak $[Ca^{2+}{}_i]$	0.001702	0.001218	0.001164	0.001085	0.001800	0.001911	0.001424	0.001592
Mean $[Ca^{2+}{}_i]$	0.000738	0.000758	0.000685	0.000459	0.000700	0.000769	0.000654	0.000614
Mean $[Ca_{SR}]$	3.081	0.701	0.786	1.805	2.556	2.925	3.501	4.103
Mean CaiT Ampl	0.001000	0.000282	0.000335	0.000372	0.000841	0.001027	0.000935	0.001155
Mean $[K^+{}_i]$	108.75	99.33	100.81	100.64	102.53	105.48	114.13	117.17
End pH_i	7.09	6.09	7.07	7.07	7.08	7.08	7.10	7.10
Mean pH_i	6.93	6.23	6.92	6.92	6.93	6.93	6.94	6.94
Mean [ADP]	0.0197	0.1448	0.7173	0.0314	0.0274	0.0235	0.0119	0.0039

Phosphometabolite Equations

During pre-ischemia, all metabolites are held at their initial values. During ischemia:

$$[\mathrm{ATP}] = 0.0006549\mathrm{t}^3 - 0.02305\mathrm{t}^2 - 0.104837\mathrm{t} + 7.216 \tag{S.1}$$

$$[\mathrm{PCr}] = -0.01259 + 12.339\mathrm{e}^{\text{-}0.92559\mathrm{t}} + 0.96819\mathrm{e}^{\text{-}0.078496\mathrm{t}} \tag{S.2}$$

During reperfusion:

$$[\mathrm{ATP}]_{\mathrm{t}} = [\mathrm{ATP}]_{\mathrm{t\text{-}\Delta t}} - \frac{[\mathrm{ATP}]_{\mathrm{t\text{-}\Delta t}} - 0.4[\mathrm{ATP}_{\mathrm{preischemic}}]}{5\mathrm{x}10^3\mathrm{ms}}\Delta\mathrm{t} \tag{S.3}$$

$$[\mathrm{PCr}]_{\mathrm{t}} = [\mathrm{PCr}]_{\mathrm{t\text{-}\Delta t}} - \frac{[\mathrm{PCr}]_{\mathrm{t\text{-}\Delta t}} - 0.75[\mathrm{ion}_{\mathrm{preischemic}}]}{5\mathrm{x}10^3\mathrm{ms}}\Delta\mathrm{t} \tag{S.4}$$

During ischemia and reperfusion:

$$[\mathrm{Cr}] = 22.2 - [\mathrm{PCr}] \tag{S.5}$$

$$[\mathrm{ADP}] = \frac{[\mathrm{ATP}] * [\mathrm{Cr}]}{[\mathrm{PCr}] * [\mathrm{H}_{\mathrm{i}}^{+}] * 1.66\mathrm{x}10^6} \tag{S.6}$$

$$[\mathrm{AMP}] = \frac{[\mathrm{ADP}] * [\mathrm{ADP}] * 1.05}{[\mathrm{ATP}]} \tag{S.7}$$

$$[\mathrm{Pi}_{\mathrm{free}}] = [\mathrm{Pi}_{\mathrm{total}} - (3 * [\mathrm{ATP}] + 2 * [\mathrm{ATP}] + [\mathrm{AMP}] + [\mathrm{PCr}]) \tag{S.8}$$

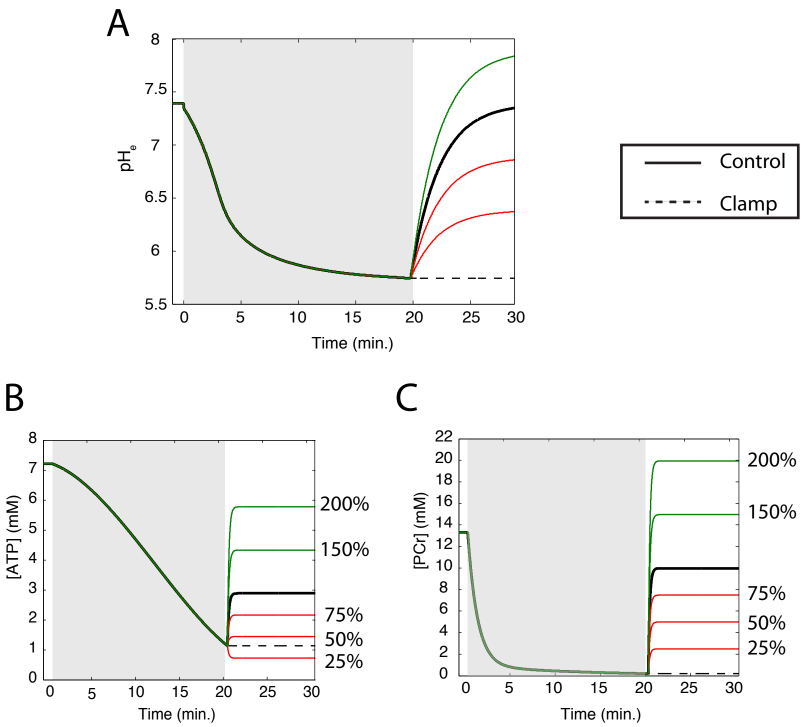

Figure S1: **Summary of simulations.** In each simulation, the end-reperfusion targets of extracellular pH (pH_e) (A) and/or ATP (B) and PCr (C) were controlled. Gray regions denote ischemic phase of simulations. See text for additional information.

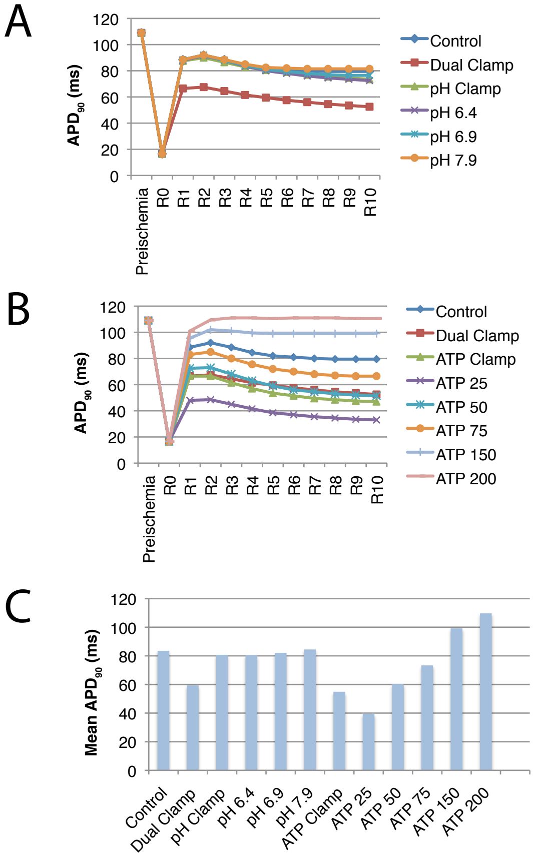

Figure S2: **Action potential duration (90) during simulated reperfusion.** The evolution of action potential duration (90) (APD_{90}) throughout reperfusion is shown for the series of simulations examining the effects of varying pH_e (A) and phosphometabolite concentrations (B). Values recorded at the end of preischemia, at the beginning of reperfusion (R0), and once per minute of reperfusion (R1-R10) are shown. The mean APD_{90} during reperfusion for each simulation is shown in (C).

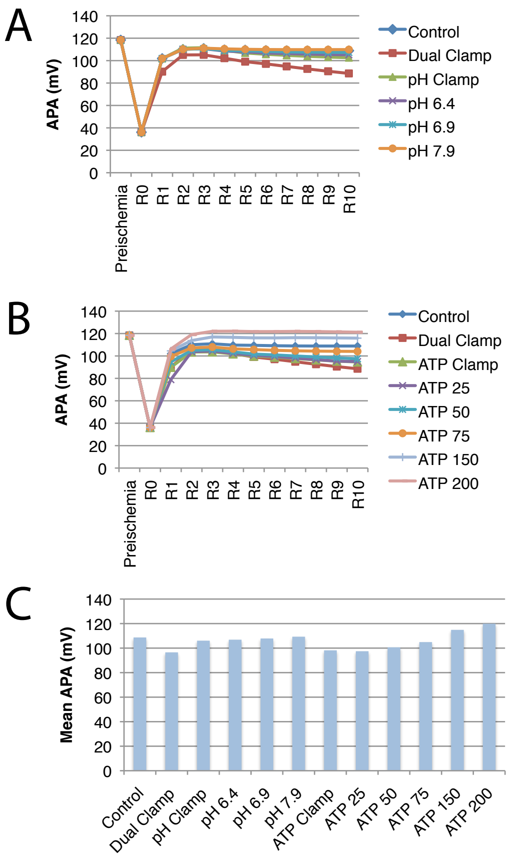

Figure S3: **Action potential amplitude during simulated reperfusion.** The evolution of action potential amplitude (APA) throughout reperfusion is shown for the series of simulations examining the effects of varying pH_e (A) and phosphometabolite concentrations (B). Values recorded at the end of preischemia, at the beginning of reperfusion (R0), and once per minute of reperfusion (R1-R10) are shown. The mean APA during reperfusion for each simulation is shown in (C).

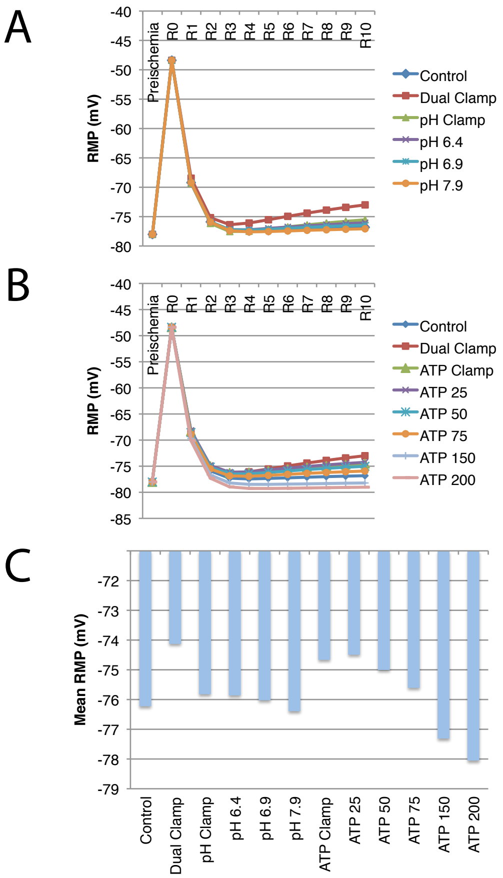

Figure S4: **Resting membrane potential during simulated reperfusion.** The evolution of resting membrane potential (RMP) throughout reperfusion is shown for the series of simulations examining the effects of varying pH_e (A) and phosphometabolite concentrations (B). Values recorded at the end of preischemia, at the beginning of reperfusion (R0), and once per minute of reperfusion (R1-R10) are shown. The mean RMP during reperfusion for each simulation is shown in (C).

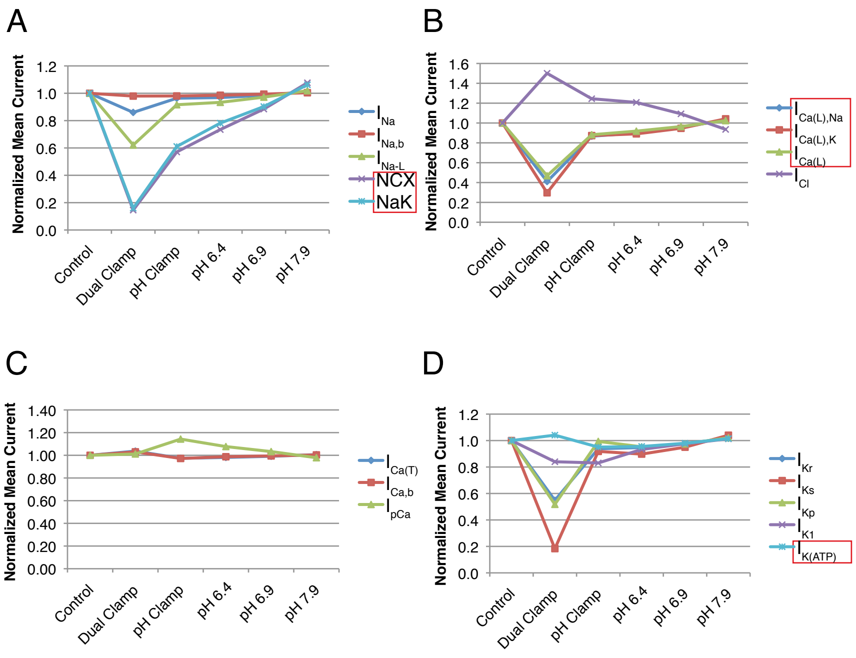

Figure S5: **Normalized mean currents throughout 10 minutes of reperfusion with variable** pH$_\mathbf{e}$ **recovery.** For each of 17 transmembrane currents, mean current in each simulation is normalized to the corresponding control. Currents that are directly modulated by pH and/or phosphometabolite concentrations are shown in red boxes.

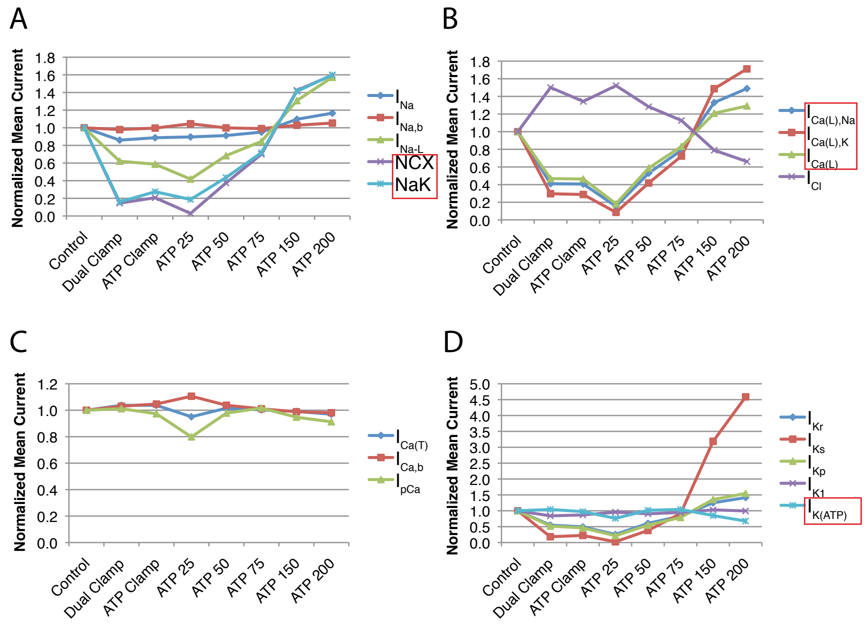

Figure S6: **Normalized mean currents throughout 10 minutes of reperfusion with variable phosphometabolite end-reperfusion target concentrations.** For each of 17 transmembrane currents, mean current in each simulation is normalized to the corresponding control. Currents that are directly modulated by pH and/or phosphometabolite concentrations are shown in red boxes.

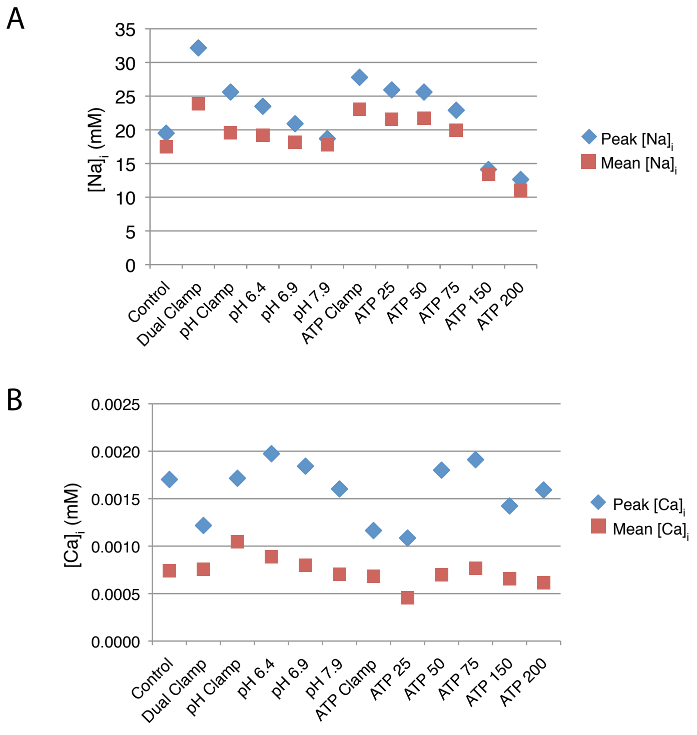

Figure S7: **Sodium and Calcium Concentrations During Simulated Reperfusion.** Peak (blue diamonds) and mean (red squares) intracellular sodium (A) and calcium (B) concentrations.

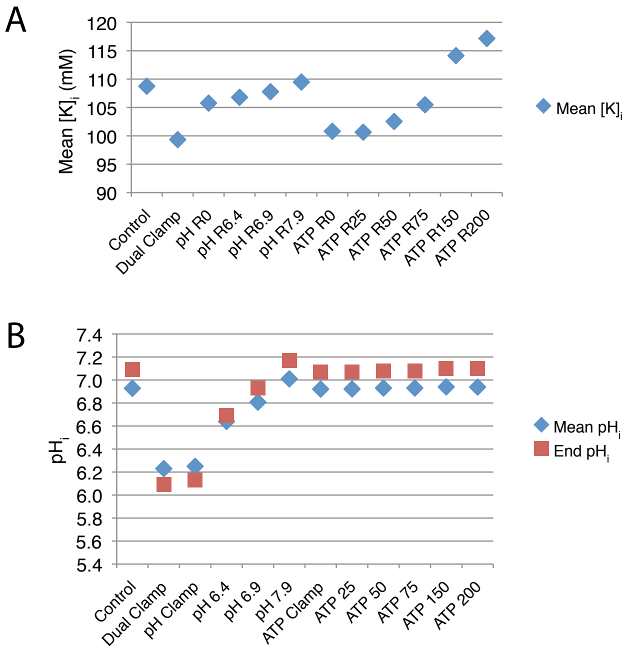

Figure S8: **Intracellular pH and Potassium Concentrations During Simulated Reperfusion.** (A) Mean intracellular potassium concentrations. (B) Mean intracellular pH (blue diamonds) and pH after 10 minutes of reperfusion (red squares).
